# The Importance of Including Non-Household Environments in Dengue Vector Control Activities

**DOI:** 10.3390/v15071550

**Published:** 2023-07-14

**Authors:** Víctor Hugo Peña-García, Francis M. Mutuku, Bryson A. Ndenga, Joel Omari Mbakaya, Samwuel Otieno Ndire, Gladys Adhiambo Agola, Paul S. Mutuku, Said L. Malumbo, Charles M. Ng’ang’a, Jason R. Andrews, Erin A. Mordecai, A. Desiree LaBeaud

**Affiliations:** 1 Department of Biology, Stanford University, Stanford, CA 94305, USA; emordeca@stanford.edu; 2 School of Medicine, Stanford University, Stanford, CA 94305, USA; jandr@stanford.edu (J.R.A.); dlabeaud@stanford.edu (A.D.L.); 3 Department of Environmental and Health Sciences, Technical University of Mombasa, Mombasa 80110, Kenya; fmutuku73@gmail.com; 4 Kenya Medical Research Institute, Kisumu 40100, Kenya; bndenga@yahoo.com (B.A.N.); ojoel58@yahoo.com (J.O.M.); ndire.samwel@gmail.com (S.O.N.); gladysagola7@gmail.com (G.A.A.); 5 Vector Borne Disease Control Unit, Msambweni County Referral Hospital, Msambweni, Kwale County 80404, Kenya; paulosillah@gmail.com (P.S.M.); saidmalumbo6@gmail.com (S.L.M.); charlesnganga069@gmail.com (C.M.N.)

**Keywords:** *Aedes aegypti*, vectorial capacity, vector sampling, households, non-household environments, simulations

## Abstract

Most vector control activities in urban areas are focused on household environments; however, information relating to infection risks in spaces other than households is poor, and the relative risk that these spaces represent has not yet been fully understood. We used data-driven simulations to investigate the importance of household and non-household environments for dengue entomological risk in two Kenyan cities where dengue circulation has been reported. Fieldwork was performed using four strategies that targeted different stages of mosquitoes: ovitraps, larval collections, Prokopack aspiration, and BG-sentinel traps. Data were analyzed separately between household and non-household environments to assess mosquito presence, the number of vectors collected, and the risk factors for vector presence. With these data, we simulated vector and human populations to estimate the parameter *m* and mosquito-to-human density in both household and non-household environments. Among the analyzed variables, the main difference was found in mosquito abundance, which was consistently higher in non-household environments in Kisumu but was similar in Ukunda. Risk factor analysis suggests that small, clean water-related containers serve as mosquito breeding places in households as opposed to the trash- and rainfall-related containers found in non-household structures. We found that the density of vectors (*m*) was higher in non-household than household environments in Kisumu and was also similar or slightly lower between both environments in Ukunda. These results suggest that because vectors are abundant, there is a potential risk of transmission in non-household environments; hence, vector control activities should take these spaces into account.

## 1. Introduction

Arboviruses (arthropod-borne viruses) are an emerging threat in African countries where the historically high burden of malaria masks the true prevalence of these diseases [1,2,3,4]. African countries mainly located in Western and Eastern Africa have recorded numerous outbreaks in recent years, with prevalence ranging from 3 to 29% [5]. One of these affected countries is Kenya, where at least three outbreaks have been recorded in the last decade [4], and there is evidence of multiple dengue virus (DENV) serotypes circulating [6,7].

Kenya’s situation has been well described in several studies conducted in Kisumu and Ukunda, with two cities located in the western and coastal regions of Kenya, respectively: studies have revealed seroprevalence in people from all ages [8,9,10,11] reaching values up to 67% [12] as well as the circulation of four DENV serotypes at high seroprevalence [7,10,12].

In addition, the presence of this vector and its traits have been described for these cities [13,14,15]. The vector responsible for DENV transmission in Kenya is *Aedes aegypti*, which usually lives in human-associated environments due to its anthropophilic feeding preference, and is mainly found in urban environments where a higher abundance of people is present [16,17]. In the absence of better and more affordable tools, current control strategies are strongly focused on the interruption of vector—human contact to avoid the transmission of this virus through strategies like the elimination of mosquito breeding places and the spraying of insecticides [18]. However, most of these control activities are heavily focused on households (HH) and are based on the assumption that mosquitoes are mostly domestic and that people spend most of their time in the household [19]. However, some studies have shown that *Ae. aegypti* mosquitoes are more active during daylight hours [14,20,21,22] when there are increased chances of people spending a relevant portion of time in places other than HH. Information related to the risk of non-household (NH) environments is scarce and poor, but some work developed in other countries has suggested a significant infection risk in places like markets [23], schools [24,25], hotels [26] or even in uninhabited or abandoned spaces [27]. Taken together, there is evidence suggesting that a significant risk of infection could occur in NH locations in urban areas, as suggested previously in other settings [28]. 

Based on the entomological information from field surveys, we aimed to describe the relative abundance of vectors in household and non-household environments in the Kenyan cities of Kisumu and Ukunda based on the entomological metric of vector-to-human density (*m*). Although this parameter cannot be taken as a direct measure for transmission risks, other metrics like vectorial capacity depend proportionally on this parameter [29,30,31,32]. Our purpose was to estimate this parameter based on data collected from intensive fieldwork and to obtain realistic data-driven estimates of the relative vector density for both HH and NH environments in these two cities to guide vector control activities. 

## 2. Materials and Methods

### 2.1. Vector Sampling and Data Collection

**Figure 1:**
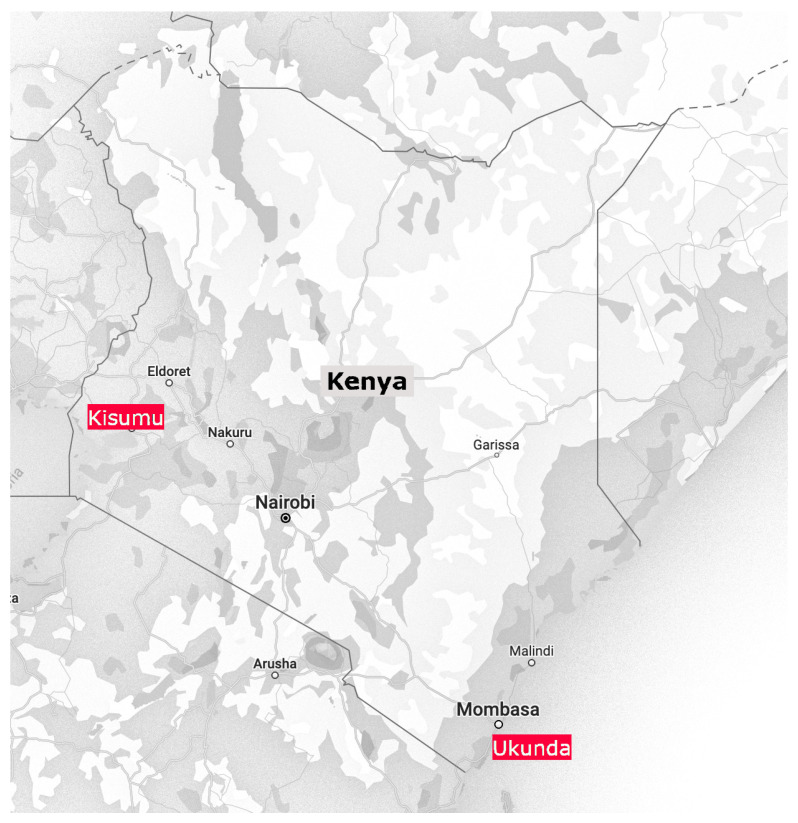
Location of study sites in Kenya.

The aim of the fieldwork was to assess the entomological risk in HH (including indoors and outdoors that are part of the domestic environment of a house) and NH environments (including indoors and outdoors that are not domestic but designated to uses other than house living) in two cities in Kenya where the active transmission of dengue has previously been established, vector populations have been characterized, and some interventions have been developed to be implemented: Kisumu and Ukunda [8,9,10,11,13,33] (Figure 1). Kisumu is the third largest municipality in Kenya, with approximately 398,000 inhabitants, according to data from a census in 2019. It is located in the western part of the country, next to Lake Victoria. Ukunda is a smaller municipality with approximately 78,000 inhabitants and is located 30 km south of Mombasa on the Indian Ocean coast of Kenya.

For sampling purposes, eight zones of 200 m × 200 m were defined across the entire urban area of each city in order to have a good geographical representation and to include equally both planned and unplanned environmental habitat strata. Each zone was subsequently divided into subzone areas of 100 m × 100 m, where sampling was conducted, including HH and NH spaces, and equal trapping was performed effort for both space types.

Between October 2020 and January 2022, four sampling strategies were carried out in the urban settings of these cities in order to collect and measure the abundance of different stages of mosquitoes: ovitraps, larval collections, Prokopak aspirators, and BioGents (BG) sentinel traps.

We collected eggs using ovitraps, which consisted of black plastic cups filled with about 350 mL of tap, boreholes, or rainwater. Cups were lined inside with a paper towel that was partially submerged. Each zone was sampled once per month with 8 ovitraps (4 for HH and 4 for NH environments), which were placed to collect the eggs for two consecutive weeks. After this period, paper towels containing the eggs were carried to our project entomology laboratories in each city, the eggs were hatched, the larvae were grown into adults, and individuals were identified as species using morphologic keys [34,35]. 

To obtain information about the mosquito’s immature stages, larval surveys were conducted every two weeks, once per month, in all zones. Potential breeding places were inspected, and their status (larval presence or absence) was recorded. Immature mosquitoes in small positive containers (those with the presence of larvae) were counted in the container, while those in large containers were counted after the water was sieved. Pupae and larvae samples for up to 10 individuals were taken to the laboratory in 10 mL conical plastic tubes and were reared to adulthood to identify species. It is worth noting that this strategy did not employ the use of traps but instead quantified larval abundance in natural breeding places. 

The adults were sampled with two trap strategies: a Prokopak aspirator and BG sentinel traps. For the Prokopak, the same locations sampled in larval surveys were used. There, spaces around HH and NH environments were sampled by progressively aspirating walls and other potential *Aedes* resting places. In each setting, a pair of trained entomology team members conducted sampling simultaneously for twenty minutes, both indoors and outdoors [14]. The collected adults were placed in cooler boxes with ice packs and were carried to the laboratory, where they were sorted and morphically identified. 

BG sentinel traps, which target primarily mosquito females seeking breeding places, were used at a rate of two per zone. BG traps were placed in the outdoor environment for five consecutive days once per month in each zone. Every 24 h, mosquito collection cups were placed into a box with ice packs and taken to the laboratory, where identification took place.

During visits, additional information was collected in order to understand the potential risk factors and explain the presence of the insects in the structure like the type of water containers present at the time of the visit, the size of the containers, the source of the water contained, the purpose of the water, how containers were covered and the presence of other animals (Table S1). In addition, for each visit to a HH, a question about the number of inhabitants was asked to consider this parameter in future analyses (see next sections). 

Finally, temperature data were collected daily at the city level using temperature data loggers (HOBO^®^), and the daily average was calculated during the entire study period.

### 2.2. Statistical Analysis

Since our main goal was to understand the relative abundance of vectors in each environment, data were analyzed according to their collection space as belonging to a HH or NH environment. In this way, the analyses of this work were divided into two phases: first, statistical analysis to understand data extracted from fieldwork, and second, field data-driven simulations to understand the relative entomological abundance in each environment separately (see next section). 

Two comparison analyses between households and non-household environments were performed with data derived from fieldwork conducted in each city: (1) the proportion of structures for each collection date in which *Ae. aegypti* individuals were caught (compared with chi-squared proportion comparisons); and (2) the number of individuals per structure (compared with Wilcoxon’s rank sum tests). 

Additionally, binary logistic regression analyses were performed on data from larval collections in order to assess the main risk factors and explain the presence of natural larval breeding places in HH and NH structures (predictor variables in Table S1).

### 2.3. Mosquito Density Simulations

**Figure 2:**
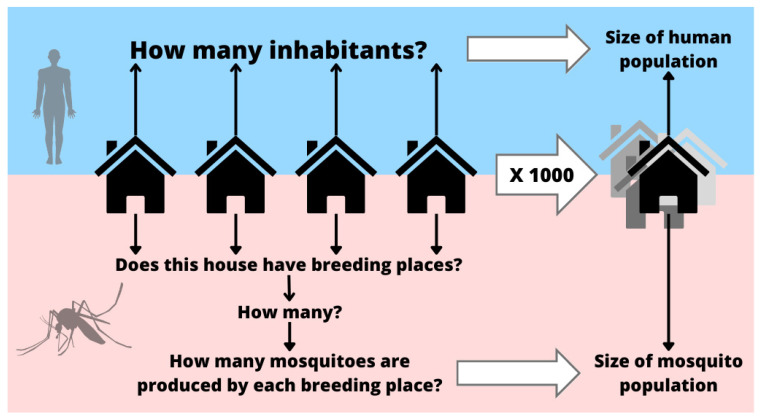
Procedure followed to generate simulated human and mosquito populations. The same human population generated across a thousand HH was also used for NH-related simulations, while a different population of mosquitoes was generated for each environment separately. In this way, we considered the differences observed between them and considered the low movement of mosquitoes.

We first aimed to simulate a distribution of likely vector abundances in HH and NH environments. To undertake this, we used the field data from each environment and city in the following way: proportions of structures with the presence of immature mosquitoes were used in a binomial distribution to generate a random number of infested structures in both environments for a thousand structures. In each of them, the number of positive containers per structure was randomly sampled from observed empirical values. For each positive container, we used distributions fitted to empirical values of immature and mature stages of mosquitoes to simulate the number of individuals. To conduct this, the number of eggs per container was randomly simulated using the appropriate fitted distribution. Assuming that some proportionality was conserved between the number of eggs laid and the number of adults that emerged, the same percentile of the number of eggs was used to estimate the number of adult individuals according to the distribution fitted to the number of adults per structure (supplementary information). Similarly, information about the number of inhabitants per house was also fitted to a Poisson distribution (Table S2). During simulations, such distributions were used to generate random numbers of inhabitants per house, and the total number of people across a thousand houses comprised the size of the human population that was used for the simulations (Figure 2). Because we assumed a very limited movement of mosquitoes, we simulated a different population for HH and NH structures separately, while a single human population was simulated based on a thousand houses. By simulating both vector and human populations, it was possible to estimate the proportion of mosquitoes in relation to humans (*m*). 

Once the number of human and mosquito individuals per house and population were calculated, we proceeded to estimate *m* in time to gain an idea of how this parameter changed during the two-year fieldwork temporal window. To undertake this, we averaged for each week the daily proportion of structures with the presence of vectors considering egg and larval stage trapping irrespective of their specific location inside the city. Following the same procedure previously described and 1000 structures for each in both environments, we simulated the number of adults and estimated the parameter *m* by considering the size of the human population.

All simulations were run 1000 times and were performed for the purpose of learning about the general shape of the distribution of *m* and its variation in time considering the two-year samplings and both environments.

## 3. Results

### 3.1. Mosquitoes Were Present and Abundant in Both Environments

During the sampling time, there were, in total, 6380 inspections performed for the presence of vectors in both cities. These were distributed through different sampling time points and strategies across both environments. Thus, for Kisumu, 3290 inspections were conducted, where 1716 resulted in the capture of individuals. In Ukunda, 1507 out of 3090 inspections resulted in the capture of vectors. 

**Figure 3:**
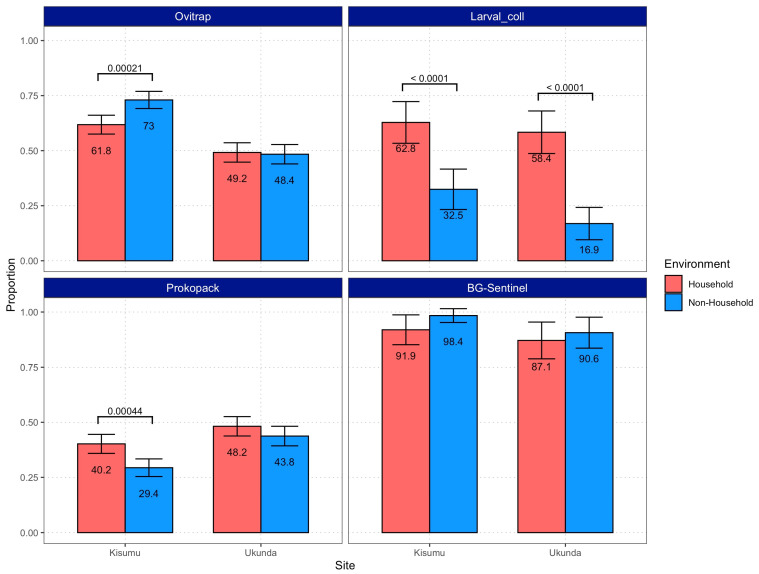
Proportion of HH and NH structures in which the presence of *Ae. aegypti* was detected through the four sampling strategies. Error bars indicate 95% confidence intervals. Values expressed as percentages are indicated in each bar, and *p*-values of significant differences (*α* = 0.05) are shown above bars.

**Figure 4:**
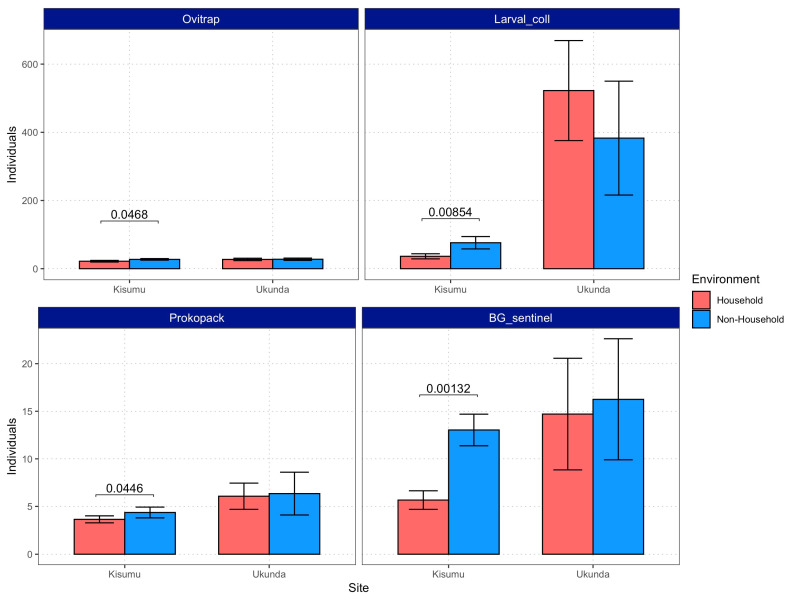
Number of individuals captured per HH or NH structure with four sampling strategies. Error bars indicate 95% confidence intervals, and *p*-values of statistically significant differences (*α* = 0.05) are shown above bars.

The proportion of structures reporting the presence of mosquitoes behaved similarly between cities but was different across the four sampling strategies. Specifically, the proportion of *Aedes*-infested structures detected through larval collections was always higher (around 2-fold) for HH environments and likewise for Prokopak samples in Kisumu. The remaining strategies resulted in either no difference or NH environments displaying a higher proportion of structures with the presence of vectors (Figure 3). On the other hand, number of individuals was consistently higher in NH environments in Kisumu, while no differences were detected in Ukunda (Figure 4).

The binomial logistic regression analyses suggested that each environment had different explanatory variables for the presence of vectors. None of the studied variables were statistically significant in predicting the variation in vector presence in NH spaces in Ukunda. In Kisumu, variables relating to clean water and domestic use in small containers were the most predictive of the vector presence in HH environments, while those relating to trash, including small plastic containers and tires containing rainwater, were most predictive in NH environments (Table 1).

### 3.2. Vector Density Is Equal or Higher in Non-Household Spaces Compared to Households

**Figure 5:**
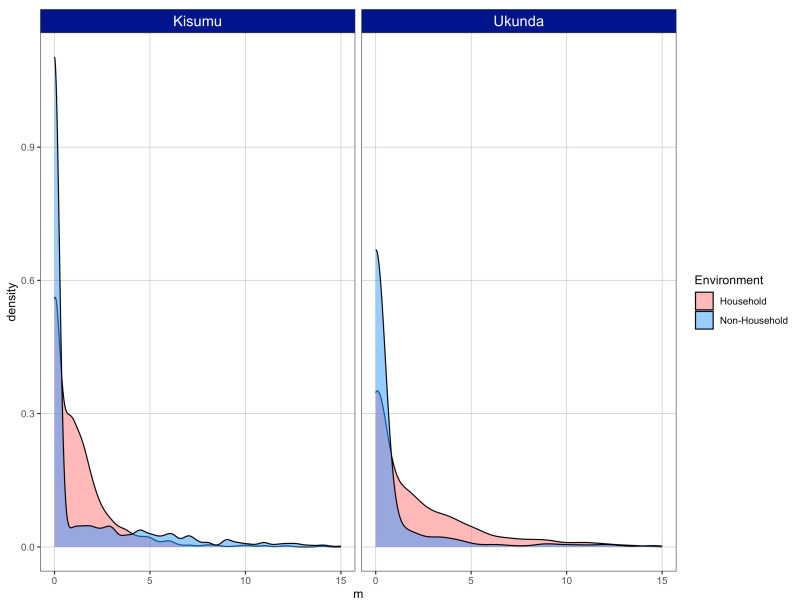
Distribution of *m* (relative-to-human vector density) for a thousand simulated structures for HH and NH environments from Kisumu and Ukunda.

From these empirical measurements of vector abundance and human population presence in each environment, we simulated vectors and human populations to estimate the density of vectors for each environment and city. The distribution of values of *m* (i.e., the relative-to-human density of vectors) differed between cities and environments: higher values were more frequent in HH from Ukunda, but the opposite occurred in Kisumu, where higher values (around four and higher) were were frequent in NH structures (Figure 5). 

**Figure 6:**
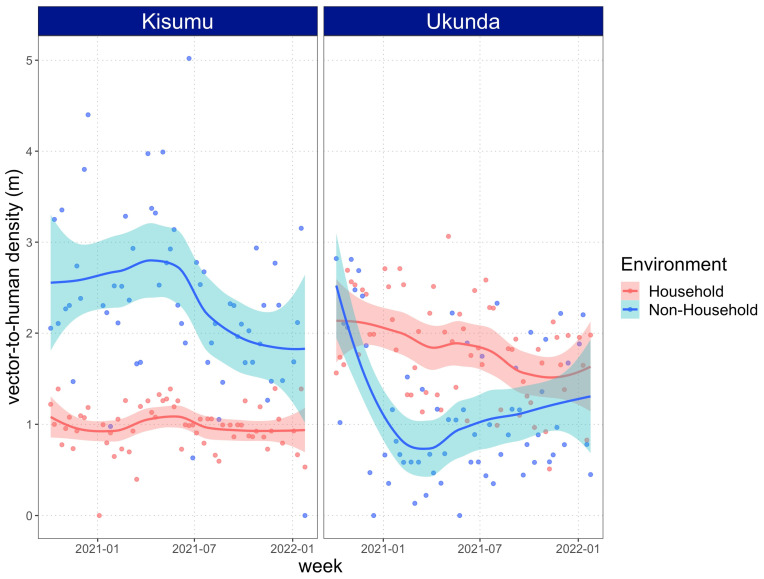
Relative-to-human vector density (*m*) simulated in time as a function of weekly averages for daily sampling data of immature stages of *Ae. aegypti.* A *LOESS* (span = 0.75) smoothed function with 95% confidence intervals was added to the plots to visualize a general trend of *m*. Plots differ in their x-axis since there were slightly different sampling times between cities.

Parameter *m* varied through time in both cities; however, NH remained consistently higher than HH environments in Kisumu. In this city, the weekly average values of *m* ranged from 0 to 5.05 (with a mean of 2.36) vectors per human in NH environments, while in HH, it ranged from 0 to 1.39 (mean of 0.972) vectors per human. On the other hand, in Ukunda, the estimation of *m* showed a more heterogeneous behavior over time. In this city, HH environments displayed higher values of *m,* but at some time periods, such as at the beginning and at the end of the study time, the confidence interval overlapped with those from NH environments. Different from Kisumu, the *m* values in Ukunda were more similar and ranged from 0 to 2.83 (with a mean of 1.15) vectors per human in NH environments and from 0.5 to 3.06 (with a mean of 1.82) vectors per human in HH environments (Figure 6).

## 4. Discussion

Arboviruses are one of the most prevalent and morbid groups of diseases in the world [36,37,38]. However, the development of control strategies other than vector control has been challenging, and some of them are still currently being evaluated in this field (like vaccines or transinfection [39,40]). For this reason, reducing mosquito density (i.e., parameter *m,* relative-to-human density of mosquitoes) has been historically the most useful strategy and is widely implemented. However, most of the current evidence and practices related to vector control are mainly focused on prevention at the HH level [19], while little attention has been put into understanding the contribution of spaces outside of the HH.

Although we observed a different pattern between HH and NH environments in these Kenyan cities, it is expected that the same phenomenon occurs in many endemic settings; therefore, further research should be conducted in each location to understand the extent of this difference. However, though the real number of infections happening in non-household spaces is hard to quantify, phenomena described in other places, like a reduction in dengue cases observed during the pandemic lockdown [41,42], the description of peak biting time during regular working hours (around 3:30 and 7:30 pm) [14], and reports on infection risk taking place in specific NH environments [23,24,25,26,27] support our results and suggest that a non-negligible number of infections are happening in those NH spaces, indicating the presence of an appropriate number of vectors.

The risk factors for the presence of immature stages of vectors align with those previously identified by Ngugi and co-authors [13]. Specifically, we found a differential ecology between environments, where those related to NH spaces were mainly associated with rainwater, tires, and trash-like containers. By contrast, HH environments were mainly associated with clean water (used for animal care and human sanitation) and the domestic use containers like pots, drums, jerrycans, and small plastic containers. The extent to which these ecological differences imply differences in control strategies should be further evaluated. Meanwhile, we believe that though insightful vector control strategies have been proposed to be applied in HH [33,43], the lack of inclusion of NH environments could lead to a latent risk that continues the transmission cycle at a city level. Unfortunately, we were unable to fit our data from the NH environment in Ukunda into a binomial logistic regression model: possibly due to excessive randomness in the distribution of evaluated risk factors, which could suggest that there are other factors involved that we could not evaluate. As a result, the specific environmental factors relating to NH environments in Ukunda remain uncertain. Unfortunately, we did not have data on other ecological variables like temperature for each environment, which would allow us to estimate differences in other transmission-related parameters like vectorial capacity or a basic reproduction number. Previous work in other locations has shown that temperature differs between indoor and outdoor spaces, which surely influences transmission [44].

It is worth noting that sampling carried out on natural mosquito populations through larval collections consistently showed a higher proportion of HH spaces with the presence of mosquitoes than NH. This result was not a direct reflection of either the proportions of structures with the presence of adults in Ukunda or adult abundance in both cities, which is also supported by simulations. This result can challenge the idea of larvae and pupae abundance as a direct representation of adult populations and, hence, transmission risk in endemic locations, which is an idea that has been previously questioned based on the results from other settings [45]. 

The same sampling effort was performed in both cities to avoid as much sampling bias as possible. We would expect sampling to be biased toward finding vectors more effectively in households compared to non-household environments since that is where sampling methods have been most developed. We did not find such systematic bias in our data because of: (1) a higher abundance of vectors in NH environments in Kisumu; and (2) heterogeneous behavior in time in Ukunda.

Our simulations assumed that the human population size was the same for HH and NH environments, which was estimated considering a HH-based number of inhabitants. However, this is likely unrealistic since it is expected that some portion of the population, like very young children and elders, spend most of their time in the HH; therefore, the value of *m* could change and subsequently affect the vectorial capacity of populations. As a result, *m* is a highly dynamic parameter that, although we attempted to capture it, depends on many other factors that are beyond the scope of our analyses.

Human mobility is perhaps the most important feature of dengue transmission that our model did not account for by focusing exclusively on the entomological component of the variation in risk between HH and NH environments. Specifically, human mobility among different urban spaces is highly important for vector-borne disease transmission and is an important area for future work. Consistently, Harrington and co-authors found that more than half of the mosquitoes which were collected in four villages in Thailand fed on people from other origins than those where the vectors were collected [46], indicating that human movement plays a significant role in transmission. For this reason, we believe that more realistic simulations involving the movement of people between both environments could capture more accurately the relative infection risk between environments as well as the true number of infections. 

## 5. Conclusions

In conclusion, our results suggest that while households are important for maintaining suitable conditions for dengue transmission, there is potential for infection risks in environments outside the household due to a similar or even higher presence of vectors in large cities. Such a presence of mosquitoes in both environments might be determined by different ecological traits in relation to breeding places. These ecological differences should be taken into account when designing vector control strategies in cities, which should include both HH and NH environments. Failing to account for the DENV risk in the NH environment may facilitate transmission even where HH control programs are highly effective. By further incorporating variation in the human population size, behavior, and mobility among environments, we could better understand the full scope of NH environments to contribute to DENV transmission as well as identify levers for effective control.

## Figures and Tables

**Table 1:** Statistically significant variables of binomial logistic regression fitted with the presence of vectors as dependent variables with a significance of 95%.

City	Environment	Factor	Coefficient	OR	*p*-Value
**Kisumu**	Household	Intercept	−2.28682	0.101589	1.64 × 10^−09^
		Type Drum	1.95693	7.07756555	0.00652
		Type Pot	2.94528	19.0159861	8.81 × 10^−05^
		Purpose Sanitation	1.37002	3.9354294	0.00899
	Non-Household	Intercept	−3.493	0.03040951	0.029187
		Size large	−28.16	5.89 × 10^−13^	0.001062
		Size medium	−25.54	8.09 × 10^−12^	7.06 × 10^−04^
		Size small	−26	5.11 × 10^−12^	7.22 × 10^−04^
		Type small plastic domestic	3.986	53.8391017	0.015713
		Type tire	5.812	334.287032	0.009137
		Source rain	26.76	4.18 × 10^11^	4.47 × 10^−04^
**Ukunda**	Household	Intercept	−2.508	0.08143094	0.002533
		Type small domestic	−4.864	0.00771954	0.003098
		Type animal feeding	−4.18	0.01529851	0.020602
		Type Bucket	−8.607	1.83 × 10^−04^	4.87 × 10^−04^
		Type Drum	−3.965	0.01896804	0.048362
		Type jerrycan	−5.016	0.006631	0.013445
		Purpose animal	3.588	36.1616802	0.011661
	Non-Household	None	NA	NA	NA

## Data Availability

Data and codes to replicate the simulations can be found in the repository github.com/vhpenagarcia/vc_kenya (accessed on 6 June 2023).

## Supplementary Materials

The following supporting information can be downloaded at: https://www.mdpi.com/article/10.3390/v15071550/s1, Table S1: Variables collected and evaluated in binomial regression model; Table S2: Distributions fitted to data from different mosquito stages data.
