# Supplementary material for: The Importance of Including Non-Household Environments in Dengue Vector Control Activities
Source: Viruses. 2023 Jul 14;15(7):1550. doi: 10.3390/v15071550 (PMC10384488; doi:10.3390/v15071550)
Supplement: Supplementary file 1 [file viruses-15-01550-s001.zip › viruses-2467164-supplementary.pdf]

# The importance of including non-household environments in dengue vector control activities

## Supporting information

Table S1: Variables collected and evaluated in binomial regression model.

| Group | Variable | Description |
|---|---|---|
| Place of container | Backyard | |
| | Open Bathroom | |
| | Drain | |
| | Flower bed | Also includes flower bush |
| | Garden | |
| | Bushes | |
| | Open kitchen | |
| | Fence | |
| | Veranda | |
| | Well | Also called borehole |
| | Corridor | |
| | Frontyard | |
| | Other | |
| Habitat size container | Small | Less than 10 liters |
| | Medium | Between 10 and 25 liters |
| | Large | Larger than 25 liters |
| Habitat/Container type | Small domestic | Tins, plastic food containers, bottles, plates, cans, cooking pots and jars |
| | Bucket | Also called basins |
| | Jerrycan | |
| | Tire | |
| | Drum | |
| | Animal feeding trough | |
| | Pot | Flower pots |
| | Treehole | |
| | Well | |
| | Other | Other type that does not fit in the main container types |
| Container covering | Completely covered | |
| | Partially covered | |
| | Not covered at all | |
| | Not possible to cover | |
| Water source | Rain | |

| | | |
|---|---|---|
| | Borehole | |
| | Tap | |
| | Stream or river | |
| | Pond | |
| | Dam | |
| | Well | |
| Water purpose | Bathing | |
| | Drinking | |
| | Cooking | |
| | Animal water | |
| | Unused or not purpose | |
| | Laundry | |
| | Sanitation | |

Table S2: Distributions fitted to data from different mosquito stages data. Distributions were checked through Kolmogorov-Smirnov goodness of fit test.

| Stage | Site | Environment | Distribution | Parameter 1* | Parameter 2* |
|---|---|---|---|---|---|
| Proportion of houses with positive containers | Kisumu | HH | Binomial | 0.628 | NA |
| | Ukunda | NH | Binomial | 0.527 | NA |
| | Kisumu | HH | Binomial | 0.538 | NA |
| | Ukunda | NH | Binomial | 0.326 | NA |
| Number of eggs | Kisumu | HH | Log-normal | 3.3 | 0.672 |
| | | NH | Log-normal | 3.4 | 0.645 |
| | Ukunda | HH | Log-normal | 3.53 | 0.797 |
| | | NH | Log-normal | 3.61 | 0.76 |
| Adults | Kisumu | HH | Weibull | 3.063 | 4.765 |
| | | NH | Log-normal | 2.045 | 0.482 |
| | Ukunda | HH | Log-normal | 2.453 | 0.668 |
| | | NH | Log-normal | 2.589 | 0.903 |

* For Parameter 1 and Parameter 2, respectively: For Binomial distribution: Probability of having positive containers. for Log-Normal distribution: mean-log and log-sd. For Weibull distribution: Shape and Scale.
